# Characterization of Novel *RHD* Allele Variants and Their Implications for Routine Blood Group Diagnostics

**DOI:** 10.3390/biomedicines12020456

**Published:** 2024-02-18

**Authors:** Eva M. Matzhold, Maria Bemelmans, Helene Polin, Günther F. Körmöczi, Marlies Schönbacher, Thomas Wagner

**Affiliations:** 1 Department of Blood Group Serology and Transfusion Medicine, Medical University of Graz, 8036 Graz, Austria; maria.bemelmans@medunigraz.at; 2 Austrian Red Cross, Blood Transfusion Service of Upper Austria, 4020 Linz, Austria; helene.polin@o.roteskreuz.at; 3 Department of Transfusion Medicine and Cell Therapy, Medical University of Vienna, 1090 Vienna, Austria; guenther.koermoeczi@meduniwien.ac.at (G.F.K.); marlies.schoenbacher@meduniwien.ac.at (M.S.)

**Keywords:** Rhesus blood group, D antigen, partial D, weak D, RHD allele, Rh diagnostics, Rhesus blood typing, RH genotyping

## Abstract

The Rh system, including the highly immunogenic D antigen, is one of the clinically most important blood group systems in transfusion medicine. Numerous alleles of the *RHD* gene are associated with variant RhD phenotypes. In case of Rh incompatibility, some of them can induce hemolytic transfusion reactions and hemolytic disease of the fetus and newborn. Thus, accurate blood group diagnostics are critical for safe transfusion therapy. We characterized phenotypes of four individuals revealing weakened D expression during routine pre-transfusion testing. Standard gel card matrix techniques with monoclonal and polyclonal anti-D antibodies were used for serological typing, complemented using D epitope and antigen density analysis. Genotyping employing PCR with sequence-specific primers, genomic and allele-specific Sanger sequencing and in silico protein analysis were performed. Four novel *RHD* alleles associated with weak D or partial D phenotypes were identified. One of the mutations is predicted to disrupt the terminal stop codon and result in an elongated translation of the mutant D protein that phenotypically exhibits a loss of D epitopes. Furthermore, a hybrid gene formed with the homologue *RHCE* gene is described. The presented data enhances the understanding of the Rh system and may contribute to continued advances in blood group diagnostics.

## 1. Introduction

The clinical importance of the Rh blood group system is mostly attributable to the D antigen, which is the most immunogenic blood group antigen in transfusion medicine. RhD is encoded by the *RHD* gene and *RHCE* is responsible for the expression of the Rh C, c, E and e antigens [1]. Numerous *RHD* gene variants are described that contribute to the high degree of diversity of the Rh system [2,3,4,5,6,7,8], often making routine blood typing a very challenging task [9,10,11,12,13,14]. Alleles associated with qualitative (partial D), or quantitative (weak D) antigen variants, are reported. The weak D phenotypes are usually caused by missense mutations in the transmembraneous or cytosolic parts of the encoded RhD polypetide, whereas most partial D variants are based on missense mutations in extracellular protein segments at the surface of erythrocytes [7,15]. As RhD and RhC proteins are determined by two homologous genes closely linked and present in chromosome 1, additionally, gene hybridization between the *RHD* and *RHCE* genes can cause partial D or rare null alleles [16]. Adding to the complexity, the presence of a C encoding allele in trans position to the *RHD* carrying allele in a heterozygous D positive genotype can weaken the expression of D, known as the “Cepellini effect”. This interference is not observed when the C gene is inherited on the same haplotype [17]. There is a high risk of developing allo-antibodies for D negative individuals following the transfusion of D positive blood. Women who are D negative and pregnant with a D positive fetus are at risk to develop allo-anti-D antibodies against their fetus’ D positive erythrocytes [18]. Rh incompatibility based on these allo-anti-D antibodies can cause hemolytic transfusion reactions and hemolytic disease of the fetus and newborn as reported in many cases. For patients with certain weak D phenotypes, including weak D type 1, 2 and 3, there is no evidence for allo-anti-D production because—albeit with low antigen density—all D epitopes constituting a complete D antigen are present [19]. Consequently, these patients can be treated with RhD positive (D+) blood. In contrast, other D variants that lack antigen parts have been reported to initiate an allo-anti-D antibody response against those epitopes that are not expressed when challenged with normal D+ erythrocytes. For patients carrying such variants, the transfusion of RhD negative (D−) blood is mandatory. In this respect, accurate Rh diagnostics of the blood receiving patient as well as of the blood donor is critical to ensure transfusion safety. However, some of these D variants may not be distinguished using serological typing methods and very weak antigen expression as caused by the rarer DEL alleles may even escape detection by standard typing. Therefore, the unambiguous identification of Rh blood group variants often requires the use of molecular methods [13,14,20,21].

Here we report the comprehensive analysis of variant D phenotypes observed in different samples during routine pre-transfusion testing. Finally, we were able to identify novel *RHD* alleles characterized by variants spreading across several exons defining weak D and partial D variants.

## 2. Materials and Methods

### 2.1. Rh Phenotyping

Standard serologic Rh antigen typing was performed on the IH-1000 automated blood group testing system (Bio-Rad) and by manual testing using standard gel card matrix techniques (Micro Typing ID System, Bio-Rad Laboratories Inc, Hercules, CA 94547 USA). The ID-Cards (Id-n°:) 5000, 5004 and 5012 based on polyclonal anti-sera and ID-Cards 5001, 5011, 5048 and 5096 with monoclonal anti-sera were applied. For screening of partial D variants, a typing kit comprising 6 different monoclonal anti-D (ID-Partial RhD Typing Set, Id-n°: 46170) was used. Indirect anti-globulin tests (IAT) with monoclonal anti-D (IAT/D) were performed to detect low levels of D antigens on the erythrocytes (Liss/Coombs 5053, Seraclone Anti-D blend). The presence of irregular antibodies in the individual’s sera also were tested with IAT. A direct anti-globulin test (DAT) was conducted to determine whether anti-erythrocyte antibodies were already coating the red blood cells.

Rh D epitope analyses were performed using 56 different human monoclonal anti-D with known D epitope specificity in LISS-antiglobulin test cards (Bio-Rad) as previously described [22].

The D antigen density of variant D and control erythrocytes was quantified via flow cytometry using five monoclonal anti-D: Brad-3, P3x290, P3x241, P3x249, and ESD1 [2]. In brief, 50 µL of the 1% red blood cells (RBC) suspension was incubated with 50 µL of monoclonal antibodies at 37 °C under repeated agitation. After two washing steps with phosphate-buffered saline (PBS), 50 µL of saturated AF488^®^ conjugated Fab Fragment Goat Anti-Human IgG (Jackson Immuno Research, West Grove, PA, USA) was added and incubated for another 30 min at room temperature followed by one further washing step with PBS. Before analysis, RBC were resuspended through a 25-gauge needle to reduce potential agglutinates. Flow Cytometry was performed using FACS Canto II and FACS Diva software (BD Biosciences, Heidelberg, Germany). D antigen density was calculated by referring median fluorescence intensities to those carried along D negative (ccddee) and D positive (CcDdee) control RBC with known D antigen density [23].

### 2.2. Molecular Genetic RH Typing

Genomic DNA was prepared from peripheral white blood cells of 350 µL of blood by magnetic particle technology with the EZI DSP DNA Blood Kit using a fully automatic DNA isolation system (EZ1, Qiagen, Hilden, Germany).

Both *RHD* and *RHCE* genotyping was performed using commercially available kits that utilize polymerase chain reaction (PCR) with sequence specific primers (SSP). Specifically, the RBC Ready Gene system CDE was used for investigating the presence of the RH D, C, c, E, e and the presence of certain partial-D alleles. Weak D and -D AddOn tested the presence of common and uncommon weak D allele variants. All the PCRs were performed according to the manufacturer’s instructions (Inno-train Diagnostik GmbH, Kronberg, Germany). The results of *RHD* Zygofast SSP-PCR specify the *RHD* zygosity (genotype): RHD*01 indicates the presence of wild type *RHD* whereas RHD*01N.01 indicates a *RHD* gene deletion.

In case of ambiguous genotyping results, sequence analysis by Sanger sequencing technique was performed. For genomic sequencing analysis, each of the 10 *RHD* exons and flanking intronic regions were amplified using *RHD* specific primers (Table 1) and SuperHot Master Mix (Bioron GmbH, Ludwigshafen, Germany). Thermal cycling was carried out with the Proflex PCR System (Applied Biosystems, Thermo Fisher Scientific Inc, Waltham, MA 02451, USA). Genomic DNA was amplified in a total volume of 25 µL, with 0.4 mmol/L primers and approximately 150 ng of DNA sample. After denaturation for 120 s at 94 °C, amplification of the *RHD* exon 1 was as follows: 35 cycles of denaturing for 15 s at 94 °C, annealing for 30 s at 64 °C and elongation for 60 s at 72 °C. The amplification of *RHD* exons 2 to 10 was based on a two-step PCR protocol with 10 cycles of denaturing for 15 s at 94 °C, annealing at 65 °C and elongation at 72 °C for 90 s both, followed by 25 cycles of denaturing at 94 °C for 15 s, annealing at 61 °C for 15 s and elongation at 72 °C for 120 s. PCR products were visualized on a 2% (wt/vol) agarose/Tris-ethylenediaminetetraacetate gel by electrophoresis.

For haplotype identification, the allele-specific separation of diploid genomic DNA by magnetic bead technology with primers detecting the nucleotide alterations c.916A (5′-GCTGGGCTGATCTCCA-3′), c.916G (5′-CTGGGCTGATCTCCG-3′), c.410T (5′-CCATCACCACCAACTGCA-3′) and c.410C (5′-CATCACCACCAACTGCG-3′) was applied as described before [24].

For the removal of excess primers and nucleotides, the amplified DNA was enzymatically treated with ExoSAP-IT PCR product clean up reagent (Thermo Fisher Scientific Inc, Waltham, MA, USA) according to the manufacturer’s protocol. DNA sequencing was completed in forward and reverse directions with AB 3500 Series Genetic Analyzer (Thermo Fisher Scientific Inc, Waltham, MA, USA) using BigDye V3.1 cycle sequencing reagents and *RHD* specific primers (Table 1). The nucleotide sequences obtained were compared to the reference sequence of *RHD* (RHD*01, NG_007494.1) under the usage of SeqScapeTM Software v.3.0. The novel alleles were either submitted to the European nucleotide database (ENA) or GenBank. Their accession numbers are indicated in the result section.

The translation of the complementary DNA (cDNA) sequence to protein sequence and in silico protein function prediction were completed using Expasy, the bioinformatics resource portal of the SIB Swiss Institute of Bioinformatics (https://www.expasy.org/), accessed on 17 November 2023 [25,26], and Polyphen-2 (http://genetics.bwh.harvard.edu/pph2/) accessed on 20 November 2023, respectively [27]. The automated protein structure homology-modelling platform SWISS-MODEL and the SWISS-MODEL Repository database (https://swissmodel.expasy.org/interactive), accessed on 5 February 2024, were used for generating a three-dimensional model of the RhD protein (Q02161, Blood group RhD polypeptide) [28,29].

**Table 1:** Primers used for amplification and sequencing of the *RHD* gene.

Primer Name ^1^	Sequence, 5′3′	Purpose	Target Exons
RHD_Ds1_Ex1_F ^2^	TCAACTGTGTAACTATGAGGAGTCAG	A	Exon 1
RHD_Ds1_Ex1_R ^2^	GCTATTTGCTCCTGTGACCACTT	A and S	
RHD_I1-1405_Ex2_F ^3^	CATTTCCCCTATTTAACAGACAAGAACAAG	A	Exon 2
RHD_I2+61_Ex2_R ^3^	GGCAATATCCCAGATCTTCTGGAACC	A and S	
RHD_I2-182_Ex3_F ^3^	AGGCCACCTTAACGGGAGAAGAG	A	Exon 3
RHD_I3+301_Ex3_R ^3^	GCTATGTTGCCCAGCTCGGTCC	A and S	
RHD_I3-45_Ex4_5_F ^3^	AAGGACTATCAGGGCTTGCCCCGTGC	A and S	Exons 4 and 5
RHD_I5+149_Ex4_5_R ^3^	CCACTGTGACCACCCAGCATCCTA	A and S	
RHD_I5+1463_Ex6_F ^3^	AGGCAGTAGCGAGCTGGCCCCTCA	A	Exon 6
RHD_I6+57_Ex6_R ^3^	GCACTGCACAGTGGCCCATCAGGTCC	A and S	
RHD_I6-160-Ex7_F ^3^	CTCTTCATTTCAACAAACTCCCCGA	A	Exon 7
RHD_I7+326_Ex7_R ^3^	TGGGAGCACGTCCACAGCAAAG	A and S	
RHD_I7-327_Ex8_F ^3^	TGGAGGCTCTGAGAGGTTGCGG	A	Exon 8
RHD_I8+151_Ex8_R ^3^	GCCTCACAGTCCACATTAGCAGCAG	A and S	
RHD_I8-67_Ex9_F ^3^ RHD_I9+62_Ex9_R ^3^	TGAGATACTGTCGTTTTGACACACAATACTTC GTTTTACTCATAAACAGCAAGTCAACATATATCCT	A and S A	Exon 9
RHD_I9-417_Ex10_F ^3^	CACTCCAGCCTGAGACAAGAGCGAAAC	A	Exon 10
DEX10SP-1358-Ex10_R ^3^	CAGTGCCTGCGCGAACATTG	A and S	
RHDSeq_Ds1_Ex1_F ^2^	TCCATAGAGAGGCCAGCACAA	S	Exon 1
RHDSeq_I1-147_Ex2_F ^3^	ATTCAGTTGAGAACATTGAGGC	S	Exon 2
RHDSeq_I2-151_Ex3_F ^3^	GAGATGGTCACTCCACTCTGTAG	S	Exon 3
RHDSeq_I4-103_Ex4_R ^3^	TGATGGAAGGGCTTCAGACACC	S	Exon 4
RHDSeq_I5-127_Ex5_R ^3^	CCTAGAGCTCCACTGTAGAGGC	S	Exon 5
RHDSeq_I5_149_Ex6_F ^3^	TCCACTGATGAAGGACACGTAG	S	Exon 6
RHDSeq_I6_130_Ex7_F ^3^	GTGCACATTCAAGTCTGAGAAG	S	Exon 7
RHDSeq_I7_121_Ex8_F ^3^	ATGTACCAGCCAGGGAGAGGAC	S	Exon 8
RHDSeq_I9-58_Ex9_R ^3^	CAAGTCAACATATATACTCAGG	S	Exon 9
RHDSeq_I9_119_Ex10_F ^3^	TCCAAGATCTCTTCCAATTCAG	S	Exon 10

^1^ Employed forward (F) and reverse (R) primers are indicated. ^2,3^ Primers were previously described by Legler et al. [30] and Gassner et al. [31], respectively. A = amplification; Ex = exon; I = intron; S = sequencing.

## 3. Results

During routine antigen typing, an unknown D status was encountered in four individuals. These unexpected results led to an additional serological and molecular work-up.

**Figure 1:**
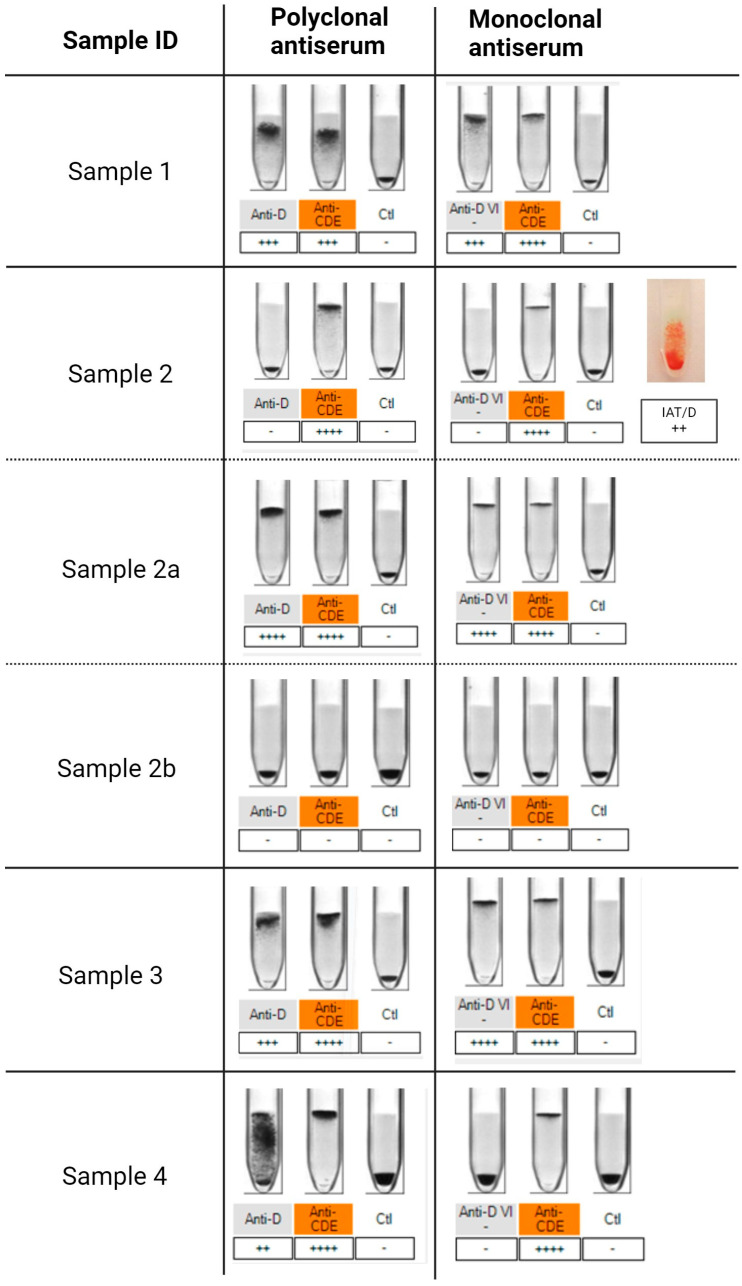
RhD and RhCE antigen phenotyping. Reagent ID-gel cards containing polyclonal (**left** panel) and monoclonal antisera (**right** panel) were used. Positive reactions are indicated by agglutinated red cells present on the top level (4+) of the gel tubes. Typing of Sample 1 indicated weakened D positive agglutination reactions (3+). Sample 2 appeared to be RhD negative, but further typing by indirect anti-globulin test (IAT/D) revealed a positive (2+) detection of D antigens. Her mother (Sample 2a) was typed as RhD positive whereas her father (Sample 2b) was typed as RhD negative. Sample 3 indicated discrepant results showing a weak agglutination reaction (3+) for D using polyclonal antiserum and a strong positive reaction (4+) using the monoclonal reagent. Sample 4’s discrepancy resulted in a weakened D expression (2+) detected with polyclonal anti-D, whereas it was detected to be RhD negative with monoclonal anti-D. Created with BioRender.com.

**Figure 2:**
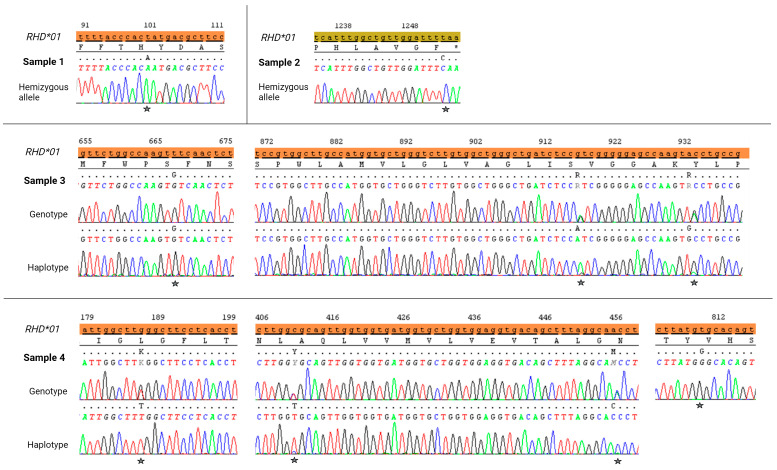
Electropherogram of *RHD* sequencing analyses. Partial nucleotide sequences of the coding region and compared to the RHD*01 reference allele are displayed. Nucleotide positions of the variants detected are indicated: Sample 1, exon 1 c.100T>A; Sample 2, exon 10 c.1252T>C; Sample 3, exon 5 c.667T>G and exon 6 c.916G>R (G>A), c.932A>R (A>G); Sample 4, exon 2 c.186G>K (G>T), exon 3 c.410C>Y (C>T), c.455A>M (A>C), exon 6 c.809T>G. Heterozygous sequences are indicated using the IUPAC code with M for A>C, K for G>T, R for G>A and A>G and Y for C>T. Created with BioRender.com.

**Figure 3:**
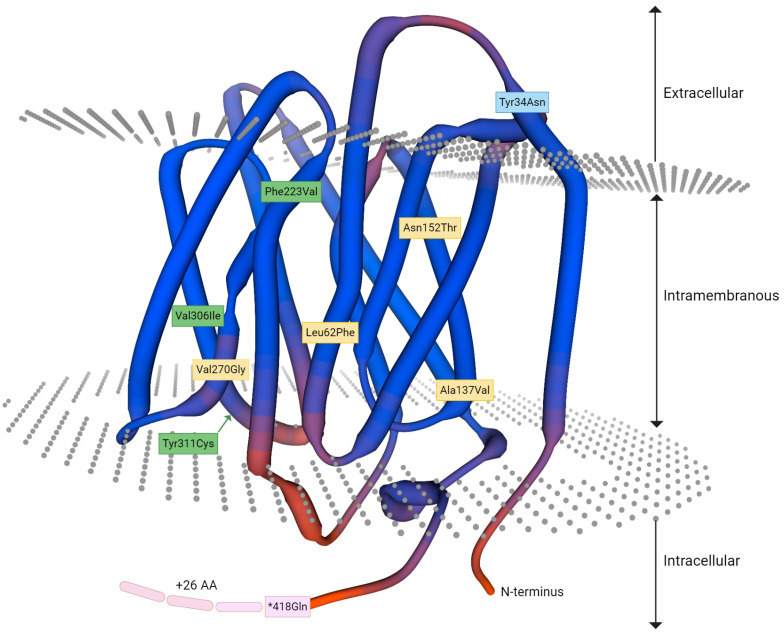
Three-dimensional model of the RhD polypeptide present in the erythrocyte membrane. The RhD reference protein structure was generated automatically by the SWISS-MODEL homology-modeling pipeline and modified to show the localization of amino acid (AA) alterations based on the variants detected in the novel *RHD* alleles. The substitutions identified in the four individual samples are indicated: Sample 1 (light blue), Sample 2 (pink), Sample 3 (green) and Sample 4 (yellow). As predicted for the sequence variant p.418Gln resulting in elongated translation at the C-terminal end of the sequence, the intracellular amino acid strand has been extended. It should be noted that this diagram does not reflect the possible structural changes introduced by each variant. Modified with BioRender.com.

The detailed results of phenotyping and molecular genetic analysis are summarized in Table 2. Serologic agglutination tests with the standard blood typing gel cards are displayed in Figure 1. The results of the sequencing analysis are shown in Figure 2. In Figure 3, the amino acid alterations encoded by the novel *RHD* alleles are illustrated.

### 3.1. Sample 1

Serologic RhD typing indicated weakened positive (3+) agglutination reactions. The D antigen density was found to be reduced at 1600 D sites per erythrocyte falling below the normal range of 10.000–20.000 D. The epitope mapping for the D antigen did not indicate a qualitative alteration, as all employed monoclonal anti-D were reactive with this sample.

Genotyping using Zygofast SSP-PCR confirmed the hemizygous presence of the *RHD* gene in the patient. CDE SSP-PCR identified the presence of a *CcDee*-associated genotype. None of the included variant alleles were confirmed by weak D SSP-PCR, therefore initiating Sanger sequencing.

Upon sequence analysis, a single nucleotide substitution c.100T>A (p. Tyr34Asn) was identified in *RHD* exon 1. The alteration encodes tyrosine instead of asparagine at amino acid position 34, suggested to be located in the extracellular segment of the RhD protein. This newly described allele was deposited into Genbank and assigned the Accession number LR736687.1. Protein analysis with Polyphen-2 predicted the impact of the amino acid substitution to alter the protein function by a high score value of 0.987.

### 3.2. Sample 2

Initially, manual Rh typing of the newborn baby showed D negative agglutination reactions together with the presence of RhC. To rule out the existence of a variant RhD further investigations using IAT/D were carried out and confirmed D positive red blood cells (RBCs) and a negative DAT.

The SSP-PCR results indicated a *CcDee*-associated genotype and hemizygosity for the presence of *RHD*.

Sequencing analysis identified the nucleotide change c.1252T>C (*418Gln) in exon 10 (Accession number LR738854.1). The variant results in the abolition of the terminal stop codon. The translation of additional twenty-six amino acids at the C-terminal of the protein was predicted by using Expasy.

With the purpose of familial analysis, samples of both the mother and the father of the newborn were investigated. Analysis of the individuals’ mother (sample 2a) showed a D positive phenotype with Cc and Ee antigen-specific markers present as detected via SSP-PCR. Additionally, the genetic analysis revealed the presence of two *RHD* alleles indicating homozygosity. Subsequent Sanger sequencing detected the heterozygous presence of c.1252T>C, which identified her as carrier of the variant she had passed to her offspring.

The fathers’ phenotype (sample 2b) was determined to be D negative which was confirmed by a negative reaction obtained with serological testing using IAT/D and by genotyping, determining the absence of *RHD* with a molecular background of RHD*01N.01/RHD*01N.01.

### 3.3. Sample 3

Standard serology showed a D positive phenotype along with the presence of RhC, c and e antigens. Minor differences in agglutination strengths for the D antigen were detected between automated and manual methods, prompting suspicion of the presence of a weakened D phenotype. Although D epitope mapping demonstrated a normal epitope profile, a diminished antigen density of 3.500 sites per erythrocyte was estimated.

Genotyping by Weak D SSP-PCR did not result in the detection of any investigated allele. Interestingly, CDE SSP-PCR was not conclusive, indicating the potential presence of two different D partial alleles. The D AddOn SSP-PCR investigating additional rare *RHD* gene variants was performed and remained inconclusive. The Zygofast SSP-PCR determined a homozygous D genotype (RHD*01/RHD*01).

Sequencing analysis showed the presence of one homozygous alteration in *RHD* exon 5 (c.667T>G, p. Phe223Val), and two nucleotide changes in the heterozygous state in *RHD* exon 6 (c.916G>A, p.Val306Ile; c.932A>G, p.Tyr311Cys), respectively.

After allele-specific separation with each c.916A and c.916G primers prior to sequencing, the two underlying haplotypes were identified: haplotype 1 exhibited c.667T>G (p.Phe223Val), defining the RHD*08 (DFV) allele. The analysis of the second haplotype revealed c.667T>G (p.Phe223Val), c.802—131A>G, c.916A (p.Val306Ile), c.932G (p.Tyr311Cys), [32] and c.939+21-24delinsTGCT. This previously unknown *RHD* allele (Accession number PP064006) is characterized by a gene conversion of *RHD* and *RHCE* between c.802-131 and c.939+24, resulting in an *RHD-RHCE* hybrid.

### 3.4. Sample 4

Serological Rh antigen typing detected the presence of RhC. D typing revealed a negative reaction with monoclonal anti-D and a weak positive reaction using polyclonal anti-D. The presence of D was confirmed by the result of the IAT/D. Partial D screening revealed negative reactions with two of six monoclonal anti-D.

*RHD* genotyping confirmed the CcDee phenotype and indicated the homozygous presence of RHD*01W.1/.1.2 (*Weak D type 1/1.2*).

Sequencing determined the heterozygous variants in coding regions of *RHD* exons 2, 3 and 6, and one homozygous variant (c.809T>G defining RHD*01W.1) in exon 6. Haplotype specific analysis under the usage of c.410T and c.410C specific primers could define a *RHD* allele (Accession number PP064005), characterized by c.186G>T (p.Leu62Phe), IVS2+51 C>T, c.410C>T (p. Ala137Val), c.455A>C (Asn152Thr), IVS3+9 T>C, IVS3+41 G>A, IVS3+69 A>G, IVS3+153 G>A and c.809T>G (p.Val270Gly). RHD*01W.1 was confirmed to be present in trans.

While Samples 1, 2, and 3 were provided by Caucasian individuals, Sample 4 was obtained from a blood donor of Black heritage.

## 4. Discussion

To prevent a hemolytic transfusion reaction of the blood-receiving patient, blood with a compatible ABO and Rh type has to be provided. It is therefore important to resolve inconclusive blood phenotyping results of patients and blood donors via extended serologic and, if available, using molecular testing.

Here we present the investigations of the samples of four individuals showing weakened D antigen expression in routine blood group diagnostics. In all these cases, the initial standard molecular genetic tests remained inconclusive, and the presence of rare D variants was suspected.

Sample 1 was identified to carry a previously unknown single nucleotide variant, resulting in an amino acid change in the first extracellular loop of the RhD protein. All the anti-D antibodies used to examine the epitope pattern reacted positively with the sample RBCs, suggesting that the D antigen was quantitatively reduced but not qualitatively altered. The extracellular location of the substitution, however, raises the possibility of this variant becoming susceptible to alloimmunization following a transfusion or pregnancy. This has been previously described for an individual carrying the DWI variant who also had a highly retained D epitope composition [33]. To monitor the future occurrences of respective irregular antibodies, a follow-up analysis is recommended.

Sample 2 initially showed negative reactions regarding the presence of D when performing standard serological analysis. The positive agglutination reaction observed with anti-CDE anti-serum and the detection of RhC led us to perform additional serological analysis to not miss the presence of a weakly expressed D antigen as described for the “Cepellini effect” [17]. Actually, IAT/D testing, which is more sensitive, revealed the D positive phenotype. Two of six monoclonal anti-D reacted negatively with the newborn’s RBCs, indicating the loss of D epitopes and the presence of a very weakly expressed D partial variant. Sequencing analysis identified a single nucleotide change that was predicted to result in the abolition of the translational stop and elongation of the amino acid sequence in the intracellular domain of the encoded D protein. The nucleotide variant may be responsible for a grossly reduced translation or protein expression. Changes in the secondary structure of the polypeptide expressed are likely. The localization of the alteration in the primary amino acid sequence is indicated in Figure 3, which should be interpreted with caution. The schematic diagram does not reflect any possible changes in conformation of the protein.

The analysis of her parents (Sample 2a, mother; Sample 2b, father) identified that the genetic alteration was inherited from her mother. Sequencing analysis detected the same mutation present in heterozygous state with the wild type RHD*01 allele, explaining her normal D+ phenotype.

Sample 3 was obtained from a female patient. The use of diverse reagents led to inconsistent D phenotyping results based on agglutination reactions of varying strengths. Interestingly, genotyping via SSP-PCR detected the presence of two *RHD* genes but failed to identify any allele. By allele-specific Sanger sequencing the well-defined partial D allele RHD*08 (DFV) and a novel RHD*08-like allele carrying two additional single nucleotide variants on the same haplotype could be determined. The *RHCE* specific sequence in intron 6 indicates it is an *RHD/RHCE* hybrid gene. These hybrids usually present themselves serologically as a partial D phenotype lacking some, but not all, distinct D epitopes [7]. The D epitope analysis of our sample did not indicate a loss of epitopes while the antigen density was reduced. Initially a weak D might be suspected. Despite this, it is likely that the novel allele encodes a partial D phenotype, at least associated with the RHD*08 defining c.667G nucleotide substitution [34]. To explain the phenotype observed, we can only hypothesize that the two partial D alleles in this sample encode proteins that interact in such a way that they complement one another to express the full epitope profile. For clarification, it would be helpful if family members hemizygous positive for the relevant variant D allele were available, but this was unfortunately not the case.

Sample 4 was from a blood donor presenting a weakened expression of D and extended phenotyping indicated qualitative alterations. Interestingly, the well-described RHD*01W.1 allele related to weak D type 1 and a second allele containing the RHD*01W.1 specific SNP, together with three additional variants previously reported to be associated with partial D phenotypes, were detected using haplotype sequencing [11,35]. The novel variant D may interfere with the expression of the well-defined weak D allele in the heterozygous phenotype, thereby decreasing and impairing overall antigen expression, and ultimately resulting in a loss of D epitopes, or at least the absence of detection of individual epitopes, as observed in the blood donors’ phenotype. In order to prove this hypothesis, in vitro studies investigating the structural D antigen composition would be required.

One must be aware that only screening for single SNPs like the weak D type 1 related c.809 T>G using PCR may lead to wrong results and even transfusion or Rh prophylaxis recommendations. Therefore, it is important to consider the serological expression. Also, whole-gene characterization or RNA transcript analysis may be useful to overcome these limitations.

Our investigations clearly demonstrate the complexity and difficulties of Rh blood group typing when D variants are present. Despite extensive phenotyping and genotyping, the results obtained remained uncertain. It is well established that SSP-PCR analysis is a valuable tool for analyzing blood groups at the molecular level. However, due to the limited SNP numbers in available SSP-PCR systems it sometimes is not successful when additional sequence alterations are present. In the present cases, allele-specific sequencing of *RHD* was effective to identify the underlying mutant haplotype resolving the serological discrepancies. By this approach, the knowledge of the complex Rh system can also be increased, which may facilitate the advancement of Rh blood group diagnostics.

In summary, four novel *RHD* gene variants associated with weak D and partial D phenotypes were identified. This enabled us to determine whether transfusion of D positive or D negative blood was most appropriate. If the blood-receiving patient is unambiguously negative for the D antigen, the treatment with D negative red blood cells is the standard therapy. When D variants are suspected, accurate diagnostics are critical to selecting compatible red cells for transfusion so that immunization is avoided while the rarer D negative blood is preserved.

## Figures and Tables

**Table 2:** RhD and RhCE typing results.

			Phenotyping		Genetic Typing		
Sample	D	Cc Ee	D Partial Typing	D antigen density/ D epitope mapping	SSP-PCRs: CDE Weak D Zygofast	Sanger Sequencing Gene variant (amino acid variation)	Interpretation and Phenotype prediction
1	3+ (5000, 5001, 5048) IAT/D: 2+	Cc ee	LHM76/55 (IgG): 3+ LHM77/64 (IgG): 3+ LHM70/45 (IgG): 3+ LHM59/19 (IgG): 2+ LHM169/80 (IgG): 3+ LDM1 (IgM): 2+	1600 D sites/erythrocyte epitope loss not evident	*CcD.ee* no weak D RHD*01/RHD*01N.01	Hemizygous *RHD* allele with c.100A (p.34Asn)	Weak D
2	neg (5000, 5001, 5048, 5096) IAT/D: 2+ DAT: neg	Cc ee	LHM76/55 (IgG): 2+ LHM77/64 (IgG): 1+ LHM70/45 (IgG): neg LHM59/19 (IgG): 2+ LHM169/80 (IgG): 2+ LDM1 (IgM): neg	N.A.	*CcD.ee* no weak D RHD*01/RHD*01N.01	Hemizygous *RHD* allele with c.1252C (p.*418Gln)	Partial D
2a	4+ (5000, 5001)	N. T.	N.A.	N.A.	*CcD.Ee* RHD*01/RHD*01	RHD*01 and *RHD* containing c.1252C (p.*418Gln)	D positive
2b	neg (5000, 5001) IAT/D: neg	cc ee	N.A.	N.A.	*ccddee* RHD*01N.01/RHD*01N.01	N.A.	D negative
3	3+ (5000, 5004), 4+ (5001, 5048) IAT/D: 1+	Cc ee	LHM76/55 (IgG): 3+ LHM77/64 (IgG): 3+ LHM70/45 (IgG): 3+ LHM59/19 (IgG): 3+ LHM169/80 (IgG): 3+ LDM1 (IgM): 4+	3500 D sites/erythrocyte epitope loss not evident	*Ccee*, D inconclusive no weak D RHD*01/RHD*01 D AddOn SSP inconclusive	RHD*08.01(DFV): c.667G (p.223Val) RHD*08.01-like (c.667G, c.916A, c.932G) and *RHCE-* specific sequences spanning from intron 5 to intron 6 (p.223Val, p. 306Ile, p.311Cys)	Possible Partial D
4	2+ (5000), neg (5001) IAT/D: 1+	Cc ee	LHM76/55 (IgG): 2+ LHM77/64 (IgG): 1+ LHM70/45 (IgG): neg LHM59/19 (IgG): 1+ LHM169/80 (IgG): 2+ LDM1 (IgM): neg	N.A.	*CcD.ee* RHD*0W.1/.1.2	RHD*01W.1 (Weak D type 1): c.809G (p.270Gly) RHD*(c.186T, c.410T, c.455C, c.809G) (p.62Phe, p.137Val, p.152Thr, p.270Gly)	Partial D

For serologic typing standard gel matrix ID-cards including 5000, 5001, 5004, 5048 and 5096 were used. Positive reactions are indicated by 1+, 2+, 3+ and 4+, depending on the strengths of the agglutination observed. Negative reactions are indicated by “neg”. RHD*01 indicates the presence of wild type *RHD* whereas RHD*01N.01 indicates a *RHD* gene deletion. Nucleotide variants in the coding region of RHD (c.) and the corresponding amino acid substitutions (p.) are indicated. Phenotypes encoded by the novel alleles are predicted. IAT/D: Indirect anti-globulin test; N.A.: Not applicable: N.T.: Not tested.

## Data Availability

All of the analyzed data is contained within the article. The raw sequence data are available on request from the corresponding author.
